# Training the next generation of translational scientists: The Case Western Reserve University Translational Fellows Program

**DOI:** 10.1017/cts.2022.390

**Published:** 2022-04-19

**Authors:** Cheryl L. Thompson, Tessianna A. Misko, Mark R. Chance

**Affiliations:** 1 Department of Nutrition, School of Medicine, Case Western Reserve University, Cleveland, OH, USA; 2 Case Comprehensive Cancer Center, School of Medicine, Case Western Reserve University, Cleveland, OH, USA; 3 Office of Graduate Education, School of Medicine, Case Western Reserve University, Cleveland, OH, USA; 4 Center for Translational Science Collaborative, School of Medicine, Case Western Reserve University, Cleveland, OH, USA

**Keywords:** Technology translation, Education, Training, Biomedical science, Entrepreneurship

## Abstract

**Background:**

An important part of biomedical research is the translation of discoveries into clinical or community applications that impact patient health. For a vast majority of clinical applications and sustainable community interventions, a time-tested way to get innovations to patients is through licensing of the technology and commercial development, often through startups. While biomedical scientists and trainees are schooled in discovery research, the processes of commercialization are foreign or intimidating. Further, many trainees will not aspire to a faculty position, and other avenues of advancement are desirable.

**Methods:**

At Case Western Reserve University, we developed and launched a Translational Fellows Program to provide such training for the community, focusing specifically on graduate students and postdoctoral fellows. The goals of this program include familiarizing our trainees with the principles of entrepreneurship, product development, and startups. This is accomplished through study of their laboratory’s technology to identify points of translational focus and to increase awareness to potentially move ideas and products toward societal impact. This program leverages much of our existing infrastructure and provides a mechanism for the prioritization of the translation of the technology as well as “release-time” to promote effort.

**Results:**

Launched in summer 2020, our first cohort had 3 of the 12 fellows launching startups based on their technology and submitting an National Institutes of Health Small Business Innovation Research (SBIR) proposal. At least 80% reported increased knowledge and confidence in five of six key translational competencies.

**Conclusion:**

We are now continuing and improving the program and searching for sustainable support to stabilize the program for a long-term productive future.

## Introduction

The apex goal of all biomedical research is to create positive impact on patient and population health. Translation of biomedical and community health discoveries is a priority of researchers, funding agencies, and technology transfer, as it is an important mission for all major research universities. At Case Western Reserve University (CWRU), we have nationally benchmarked programs for optimizing technologies, including general accelerators for medical devices and drugs ^1^and a translational vertical for cardiovascular discoveries [2]. On the other hand, CWRU’s graduate and clinical education, similar to most nationally, has not emphasized understanding this process, focusing rather on project management, manuscript preparation, and grantsmanship in the former, and clinical competencies in the latter. This emphasis has left us with a generation of trainees, now physicians and scientists, with limited knowledge of this area.

Some universities offer courses in translation, and topics key to the field, such as intellectual property (IP) law, the Food and Drug Administration (FDA) regulatory process and/or customer discovery, and CWRU is no exception. However, adding a complete suite of classes to an MD or PhD program could delay graduation, add expense and/or may not seem relevant to the student. On the other hand, graduate students at CWRU are increasingly entering our doctoral programs with an interest in nonacademic careers. Academic research jobs for graduating doctoral students are waning and reports by the National Institutes of Health (NIH) and the Oak Ridge Institute for Science and Education show that only a minority of STEM doctoral graduates will ultimately hold an academic research job [3]. Thus, training for nonacademic careers must be an expanding priority of biomedical graduate education.

## The Case Western Reserve University (CWRU) Translational Fellows Program

At Case Western Reserve University (CWRU), we developed the CWRU Translational Fellows Program (TFP) to help alleviate these challenges. This program was inspired by an examination of the Massachusetts Institute of Technology’s TFP [4] and Stanford’s BioDesign program[5] but was developed to reflect the needs and resources available at CWRU. We were attracted by the in-depth nature of the BioDesign program and in fact, CWRU has modeled a similar program, NeuroDesign, on BioDesign. That program required full-time support for well-advanced trainees, and thus had costs for two fellows of over $150,000 before program administrative expenses and faculty teaching time is factored in. However, the outcomes are likely significant translation of the selected technology with licensing and/or startup formation. On the other hand, the MIT program which “buys” a day a week of support can support five times the numbers of trainees. In this program, the postdoctoral student and their laboratory’s technology are selected as a “bundle” and the program is focused on helping move that technology to market. As many MIT technologies are software or “tech-related,” many can be moved to the market quickly. To adapt the best features of these programs to the CWRU environment, we recognized that locally, we had a large supply of graduate students (relative to postdoctoral students) eager to engage industry, and most of the interesting technologies in the laboratories of these students are in the biosciences, and not likely to be close to market. Thus, it is unlikely that many would be commercialized within a year.

With these factors in mind, the CWRU TFP was initiated through the Center for Translational Science Collaborative (CTSC) at CWRU. The CTSC is the hub for all translational research in Cleveland, allowing collaboration with trainers, trainees, and projects from around the university and its affiliate hospitals. We articulate five goals for the program outlining the potential benefits for all the stakeholders in Table 1.

**Table 1.** Case Western Reserve University (CWRU) Translational Fellows Program Goals

Provide an experiential learning program for biomedical science trainees to learn about the translation of discoveries into commercially viable applications
To accelerate startups and licenses of CWRU biomedical technology.
To move the needle in the culture among our biomedical research faculty to think toward translation and commercialization.
To help the next generation of biomedical sciences decide if a career in biotechnology entrepreneurship is right for them.
To increase the diversity of local biotechnology startup leaders.

The program is built as an experiential learning model where trainees use the technology from their lab as a starting point and example to learn what it would take (in general) to translate a basic discovery toward impacting patients. Our target fellows are senior doctoral students (ideally in their last year of their PhD program) and postdocs. Although we are open to different disciplines (e.g. biomedical engineering, biochemistry, etc), the fellows must be a biomedical or health-oriented PhD student or postdoc. Since a majority of these students/postdocs are supported on research grants for 40 hours/week, we provide 20% salary support for these fellows with the expectation that they will devote 20% of their time to the program for a full 12 months. This allows the fellows to continue their basic and or community research 80% of the time. Application of the content to the lab’s technology or idea has mitigated principal investigator (PI) concerns that participation in the program will slow progress toward the degree, which is a concern particularly for our MD/PhD students who are on tight timelines or otherwise impede with completion of laboratory goals. We chose an experiential learning model as the main pedagogical framework for three reasons. First, our graduate students were asking for more experiential learning opportunities (data not shown). Second, experiential learning has been shown to be a highly effective way to create lasting understanding of knowledge [6]. Lastly, by focusing on technology in their laboratory, we felt their knowledge gained would be better embraced by their PI and/or other lab members.

The TFP involves 12 months of didactic thematic instruction, connecting the fellows to each other and the TFP leadership while gaining knowledge about themes that are intended to be applied to their technology. These themes include IP and technology transfer, identifying your market potential, identifying sources of funding, and pitching your technology. Content is delivered via a variety of seminars, workshops, electronic content/resources, and mentoring within our 90-minute monthly meeting, as well as occasional additional longer workshops. Additional content is provided through asynchronous online lectures and other content. Thus, during their year, the fellows will identify the market potential of their technology, understand opportunities and hurdles with respect to IP, and perform customer discovery on their technology to identify their value proposition and be able to pitch their technology, at a minimum.

One of the most important parts of translating biomedical technology is understanding how your technology fits into the current marketplace and who will be your customer. To help our fellows understand customer discovery, we incorporated I-Corps@NCATS into the TFP. I-Corps@NCATS is a 5-week course, developed by the National Center for Advancing Translational Sciences (NCATS), based on the successful National Science Foundation I-Corps and I-Corps at NIH Entrepreneurial Training Program, both of which provide training in business modeling and the customer discovery process [7]. NCATS’s goal of incorporating I-Corps best practices in a condensed way was to improve the health of communities by speeding up the process of moving new discoveries out of the research labs and into treatments for patients. The best way to streamline this process is to help academic researchers understand how innovation is brought to market and what steps can be taken to accelerate this process.

Understanding that development of the individual leading the technology through the innovation process is just as important as the innovation itself, we make the professional development of our fellows an important part of this fellowship, weaving it throughout the fellowship programing. Toward this end, fellows participate in the CWRU Venture Mentoring Program (CVMP), also housed in the CTSC. CVMP was developed based off the MIT Venture Mentoring Service (VMS), providing team-based mentoring to our translational scientists. The goal of CVMP is to focus on the professional development of our scientists and train them how to think like an entrepreneur so that they can better translate their innovation out of the lab. There are several pillars of CVMP that pave the way for the success of our young entrepreneurs: team-based mentoring, confidentiality, and administrative support. Team mentoring gives the trainees many different perspectives, sets of advice, and breadth of knowledge that help the trainee make more informed decisions for both themselves and their technology. Team mentoring also provides trainees a chance to build relationships and grow their own networks that will be very important for their success as entrepreneurs. In CVMP, each mentee is assigned a team of 2–3 mentors, who are recruited from our mentor pool of volunteers from a variety of backgrounds and expertise related to biomedical entrepreneurship. A key component of the mentoring in CVMP is the establishment of a strong culture of confidentiality. Efforts are consistently made to maintain an environment of confidentiality, not only to protect the innovation but also to build a safe space for the entrepreneurs to share all aspects of their entrepreneurial journey (i.e. technology successes and failures, funding obstacles, venture team issues, IP frustrations, etc.) so that they can receive the support and most tailored advice for how to approach any road blocks they are facing. All CVMP mentors are paired with mentees where there is no vested interest in the technology that would inhibit unbiased mentoring. To make the most of both the mentees and the mentor’s time, our CVMP staff oversees all administrative tasks including meeting scheduling, maintaining records, and communications. Removing this burden from our participants assures that their valuable time is spent with focusing on the mentee and their project.

Aside from content, the program aims to connect fellows with resources available to them that they might not be aware of. From identifying NIH-sponsored workshops for Small Business Innovation Research (SBIR) preparation to introductions to future business partners to providing access to CWRU’s think[box], where they can build a prototype or speak to a mentored law student in the IP venture clinic [8], there are no shortages of resources at CWRU or available publicly. The issue lies with knowing where to look and what you need to know.

## Experiences and Outcomes of the First Cohort of TFP

CWRU TFP was launched in the summer of 2020 in the midst of the pandemic with all non-research meetings held remotely until summer 2021. Twenty-one applications were submitted for seven advertised slots. All applications were reviewed by all members of the leadership team and scored on a five-point scale based on potential of the applicant, interest in a career in scientific translation, and (somewhat less important) the strength of their technology. With a last-minute awarding of an internal grant, we were able to onboard additional fellows, bringing our total to 12 fellows for year 1. In year 2, we had even greater interest in the program (25 applications) and we are currently supporting nine fellows.

Our first 12 fellows represented much of the diversity that can be found here amongst our researchers. These 12 fellows brought technologies from novel therapeutics to medical devices to community-based healthcare. The stage of their careers ranged from graduate students to more senior research associates. Many fellows were interested in careers in startups and entrepreneurship, some in academic careers that had a translational focus, and some were still exploring career paths.

In addition, our fellows had a diversity of demographics and backgrounds (Table 2). Three of the fellows are underrepresented minorities and six were female. Five of the 12 fellows were senior doctoral students, 1 was a PhD-level project manager, and the remainder were postdocs. Their fields of study varied widely and included genetics, cancer biology, community-based research, systems biology and bioinformatics and biomedical engineering, with biomedical engineering being represented by about half the fellows. Amongst the fellows, the level of experience with entrepreneurship also varied from having little idea of what entrepreneurship really looks like to having some experience with I-Corps or even starting a company.

**Table 2.** Demographics of Applicants and Selected Fellows

	Applicants	Fellows
Gender		
Male	14 (67%)	6 (50%)
Female	7 (33%)	6 (50%)
Race		
White	11 (52%)	5 (42%)
Asian	6 (29%)	4 (33%)
Black	1 (5%)	0 (0%)
Hispanic	3 (14%)	3 (25%)
Standing		
Doctoral student	6 (29%)	5 (42%)
Postdoc	9 (43%)	5 (42%)
Clinical fellow	1 (5%)	1 (8%)
Other	5 (24%)	1 (8%)

As of 2 months post-completion of the first cohort, three of the fellows have started companies. All of these three have submitted an SBIR and one was successful on their first attempt. While ¼ of our trainees achieving a startup may seem modest compared to the MIT[4] or Stanford[5] models as outlined above, the latter is a 100% support program for a relatively advanced trainee and technology in the biomedical sciences, while the former is also a 20% support program, but for postdoctoral students in tech spaces. Our program is only 20% effort and is predominantly graduate students in the middle of their training. In fact, we are very proud of this rate as traditionally at CWRU, and this demographic of trainees would have a < 5% rate of company startup. Further, it is important to keep in mind that, unlike other programs, the technology need not be close to ready for commercialization. Indeed, understanding that your technology needs more development prior to translation and commercialization is a very valuable lesson for the fellows and their PIs, and one way to change the culture is by helping PIs think about this earlier in their technology development process.

An exit survey was conducted on the first cohort, with one follow-up reminder email. Ten of the 12 fellows completed the survey. The fellows felt their knowledge in the six key areas included in the survey were improved as a result of the program (Table 3). Five of the six topic areas had 80% or more of the fellows report an increase in knowledge and/or understanding, with IP being the outlier with only 60% reporting increased knowledge. Results of these targeted questions as well as open-ended questions will help us modify the curriculum for future cohorts. Based on the responses of the open-ended questions, it was clear that each fellow benefitted most from different parts of the program – some called out specific content, others the mentorship, and others the networking. Although a goal of this program was to expose our trainees to a nonacademic career path, our goal was not to necessarily get them out of academia. Indeed, a future career as an academic researcher with a translational lab would be a huge success. However, as the trainees are still completing their training, only long-term follow-up will allow us to capture true success of the program. Based on the startups created and survey results, we feel a vast majority of the fellows, after only 1 year of a 20% effort experiential learning program, will use their knowledge in their future career.

**Table 3.** Exit survey from initial cohort of Case Western Reserve University (CWRU) Translational Fellows Program

	Responses (N, from a total of 10)		
Did the CWRU TRP help improve your understanding of:	Did not help	Helped somewhat	Helped significantly
The market for my technology	1	5	4
Understanding hurdles to uptake of my technology in the real world	1	4	5
Intellectual property	4	2	4
Funding sources for commercialization	2	3	5
The FDA process	2	3	5
Pitching your technology to investors	2	2	6

One of the goals of the TFP was to help to change the culture of our faculty to be more translational-oriented. Although we do not have objective data on this, anecdotal evidence from a couple of unsolicited feedback from the PIs do suggest that the fellows bring back their learning to their lab and/or that it has influenced how they think about their technology.

## Challenges and Future Plans

As with all programs, this one is not without challenges. One of our metrics is diversity of our fellows. We have had some success but are still far from ideal, as a goal will be to have fellow representation from a cohort that is representative of our student body. In addition, our leadership team of six individuals is entirely White and has only one female, and there is a constant need to identify a sufficient number of minority and female mentors through CVMP. Although there is not one of our originally intended metrics, in our first cohort, only three of the fellows were in labs of women PIs. In year 2, none of the fellows are in labs with women PIs. Representation is important, and ensuring all PIs and students consider themselves potential innovators and entrepreneurs is necessary. Others have highlighted the need for programs like TFP and I-Corps to help bridge this lack of diverse talent in biotechnology [9].

Another challenge is that fellows in this program come in with very different levels of experience and with very different needs. Thus, the program is, by design, very flexible to allow advanced students to explore beyond what is covered in the base program through mentoring and connecting with other resources. However, this remains a challenge and was one of the noted areas for improvement on the exit survey, as content will be basic for the more experienced fellows.

Although much less cost-prohibitive compared to full-time fellowship programs, the TFP still requires a significant budget to operate. Salary support for each fellow as well as faculty effort and administrative support add up quickly. However, we believe that the returns well outweigh the investment. CWRU invested over 150K initially to get the program started. Having training grant-supported participants can help cover some of the salary. In fact, we have included this program as an option on at least two T32 applications submitted in 2021 (currently in review). We are also working with our development office to identify donor support for this program.

There are many future plans to grow and expand this program. Not all students and postdocs are in labs where technology is ready for translation and/or have a PI who is translation-oriented. Conversely, not all labs with emerging technology have a lab member interested in a career path in translational science. We are considering a program for 100% supported postdoctoral fellows where, instead of devoting 20% entrepreneurial efforts on the research they are doing in their lab, they will be brought on to exclusively participate in the program and be assigned several technologies from the CWRU technology transfer office to work through the TFP.

## Conclusions

The large number of applications to the TFP highlights the demand and need for this program at CWRU. Although still a program is in development, outcomes from our initial cohort are very promising and we are confident that we will see a strong return on our investment. In addition, the deliberate training in a nonacademic career will help us attract strong doctoral students interested in translation and innovation.
